# Analysis of *nif*H‐RNA reveals phylotypes related to *Geobacter* and Cyanobacteria as important functional components of the N_2_‐fixing community depending on depth and agricultural use of soil

**DOI:** 10.1002/mbo3.502

**Published:** 2017-08-01

**Authors:** Priscila A. Calderoli, Mónica M. Collavino, Filipe Behrends Kraemer, Héctor J. M. Morrás, O. Mario Aguilar

**Affiliations:** ^1^ Instituto de Biotecnología y Biología Molecular (IBBM) Universidad Nacional de La Plata‐CONICET La Plata Argentina; ^2^ Instituto de Botánica del Nordeste (IBONE) Facultad de Ciencias Agrarias Universidad Nacional del Nordeste‐CONICET Corrientes Argentina; ^3^ Cátedra de Manejo y Conservación de Suelos Facultad de Agronomía Universidad de Buenos Aires Buenos Aires Argentina; ^4^ INTA‐CIRN Instituto de Suelos Hurlingham, Provincia de Buenos Aires Argentina; ^5^ Instituto de Biotecnología y Biología Molecular (IBBM) Universidad Nacional de La Plata‐CONICET La Plata Argentina

**Keywords:** Cyanobacteria and *Geobacter*, *nif*H transcripts, soil diazotrophic community, soil management practices

## Abstract

In this survey, a total of 80 787 reads and 28 171 unique NifH protein sequences were retrieved from soil RNA. This dataset extends our knowledge about the structure and diversity of the functional diazotrophic communities in agricultural soils from Argentinean Pampas. Operational taxonomic unit (OTU)‐based analyses showed that *nif*H phylotypes related to *Geobacter and Anaeromyxobacter* (44.8%), *Rhizobiales* (29%), Cyanobacteria (16.7%), and *Verrucomicrobiales* (8%) are key microbial components of N_2_ fixation in soils associated with no‐till management and soil depth. In addition, quantification of *nif*H gene copies related to *Geobacter* and Cyanobacteria revealed that these groups are abundant in soils under maize–soybean rotation and soybean monoculture, respectively. The correlation of physicochemical soil parameters with the diazotrophic diversity and composition showed that soil stability and organic carbon might contribute to the functional signatures of particular *nif*H phylotypes in fields under no‐till management. Because crop production relies on soil‐borne microorganism's activities, such as free N_2_ fixation, the information provided by our study on the diazotrophic population dynamics, associated with the edaphic properties and land‐use practices, represents a major contribution to gain insight into soil biology, in which functionally active components are identified.

## 1 INTRODUCTION

Agricultural management in the extensive Pampa and Chaco plains of Argentina relies on no‐till farming (source: Aapresid, www.aapresid.org.ar) that accounts for about 27 million hectares, with an increasing simplification in crop sequence based mainly on soybean monoculture (Durán, Morrás, Studdert, & Liu, 2011; Viglizzo et al., 2011). Several adverse effects have been associated with this simplification such as lower organic carbon (OC), lower aggregate stability, modification of soil porosity due to a diminution of its total volume, as well as higher bulk density and development of platy structures (Chagas, Santanatoglia, Castiglioni, & Marelli, 1995; Novelli, Caviglia, & Melchiori, 2011; Novelli, Caviglia, Wilson, & Sasal, 2013; Sasal, Andriulo, & Taboada, 2006). Hence, the way soils are used in agriculture can deeply modify their architecture constitution (Pierce, Fortin, & Staton, 1994) with significant consequences on the soil structure and the soil–water interactions (Castiglioni, Behrends Kraemer, & Morras, 2013; Raynaud & Nunan, 2014; Reynolds, Bowman, Drury, Tana, & Lu, 2002). Moreover, crop sequence in addition to no tilling also impacts pore size as well as pore stability (Castiglioni et al., 2013; Novelli et al., 2013) with strong depth influence due to the high stratification of soil properties in this system (Cavenage et al., 1999; Wander & Bollero, 1999). It has been shown that these physical soil properties affect activity and survival of fungal and bacterial communities (Hattori, 1988). Furthermore, it has been suggested that microscale spatial patterns and depth may have a regulatory effect on bacterial density (Nunan, Wu, Young, Crawford, & Ritz, 2003) and activity (Darrah, White, & Nye, 1987; Grundmann et al., 2001).

The soil microbes play key roles in recycling carbon, nitrogen, and other nutrients as well as solubilization and uptake of P and K. In particular, biologically available nitrogen in soil is the major common nutrient that limits productivity in terrestrial ecosystems, and its input is predominantly mediated by biological nitrogen fixation (BFN) (Gaby & Buckley, 2011). BFN, consisting of the N_2_ enzymatic reduction into ammonia by nitrogenase activity, is an exclusively prokaryotic metabolic process which is performed by a phylogenetically diverse group of microorganisms belonging to *Bacteria* and *Archaea* domains (Zehr, Jenkins, Short, & Steward, 2003). This process accounts for approximately 128 million tons of nitrogen per year and is considered the main route by which fixed nitrogen enters the biosphere by natural processes (Galloway et al., 2004). Here, two major pathways for BNF have been shown either living as free‐living forms or in symbiotic/associative relationships (Izquierdo & Nüsslein, 2006; Young, 1992). The ecological significance of free‐living diazotrophs in terrestrial ecosystems can be difficult to constrain as estimates for N fixation by these microorganisms can vary widely, ranging from 0 to 60 kg Ha^−1^ per year (Cleveland et al., 1999; Hsu & Buckley, 2009; Reed, Cleveland, & Townsend, 2011).

Examination of the *nif*H gene that encodes the nitrogenase reductase subunit has been used to assess the phylogeny, diversity, and abundance of diazotrophic communities (Zehr et al., 2003). Surveys of *nif*H sequences in soil have reported important contributions which in turn have increased our understanding about N_2_‐fixation potential and dynamics in this environment (Hamelin, Fromin, Tarnawski, Teyssier‐Cuvelle, & Aragno, 2002; Niederberger et al., 2012; Rösch, Mergel, & Bothe, 2002; Wang et al., 2016; Yeager et al., 2004). Several studies emphasize that a great part of the diazotrophic diversity corresponds to uncharacterized and uncultured soil bacteria (Duc, Noll, Meier, Bürgmann, & Zeyer, 2009; Gaby & Buckley, 2011; Hsu & Buckley, 2009). Recent next‐generation sequencing techniques broaden the database of potential diazotrophs in several natural environments such as sea water, soils, and saline mats (Collavino et al., 2014; Farnelid et al., 2011; Wang et al., 2013; Woebken et al., 2015).

However, there is still considerable uncertainty about which components of the diazotrophic community are actively expressing nitrogenase in the different ecosystems. Studies which lead to the examination of soil functioning in real‐world field conditions are limited. McInnes, Shepard, Raes, Waite, and Quigg (2014) applied in situ hybridization assays to estimate BNF in ocean waters. Buckley, Huangyutitham, Hsu, and Nelson (2007) used a procedure based on ^15^N‐stable isotopic probing with soil samples to characterize and identify diazotrophs that are functionally active in situ. An alternative approach is to directly investigate the mRNA of *nif*H by RT‐PCR, which has been widely applied in aquatic environments (Bird & Wyman, 2013; Church, Short, Jenkins, Karl, & Zehr, 2005; Short & Zehr, 2007; Turk et al., 2011). Nevertheless, studies on *nif*H‐cDNA are in general, very limited in soil samples (Hsu & Buckley, 2009; McGrath et al., 2008; Niederberger et al., 2012).

We have previously reported the abundance, diversity, and structure of diazotrophic communities in a gradient of Argentinean no‐till agricultural soils under contrasting management. In that research, we used deep pyrosequencing‐based analysis of the *nif*H gene in the DNA extracted from soil samples. This investigation has revealed not only novel nitrogen‐fixing organisms but also has begun to shed light on how diazotrophic communities are effected and influenced by soil chemistry and land use (Collavino et al., 2014).

The aim of this study was to assess the effect of different agricultural managements and soil depth on the diversity and structure of the active N_2_‐fixing community. To this end, we have applied a pyrosequencing‐based analysis of *nif*H sequences obtained from soil RNA from Pergamino fields located in the Argentinean Pampas. Our results have revealed a great diversity and a high representation of sequences related to *Geobacter* and *Rhizobiales*, and also to a major group of deep divergent sequences related to Cyanobacteria. The abundance of these particular groups, the diversity of the active diazotroph community, and distributions of key populations were affected by no‐till practices and by some physical–chemistry features of the soil. These taxa that were found to be associated with characteristics of porosity and soil pore size can provide useful criteria to assess the quality of agricultural soils.

## 2 EXPERIMENTAL PROCEDURES

### 2.1 Soil sampling and nucleic acid extraction

Free N_2_‐fixing communities were studied at the agricultural area of Pergamino in Buenos Aires Province (33°56′36″S; 60°33′57″W) from two soil depths of 0.0–10 cm (surface) and 10‐20 cm (subsurface) in June 2012. Soil samples of this site were previously studied by our team (Collavino et al., 2014). The studied soil is Typic Argiudoll, silty loam found in the surface horizon, and silty clay loam in the deeper layers (Duval et al., 2013). The clay mineralogy consists of 2:1 clays, mainly illites with a small proportion of irregular interstratified illite–smectite minerals, and traces of kaolinite.

Two different no‐till agricultural managements were examined as treatments. Good agricultural practices (GAP) are characterized by intensive crop rotation (soybean–maize), practice of winter cover crops, nutrient replacement, and low agrochemical use (herbicides, insecticides, and fungicides). Poor agricultural practices (PAP) are characterized by crop monoculture (soybean), low nutrient replacement, high agrochemical use, and lower yields. Natural environment (NE) was used as treatment reference, and consisted of natural grassland close to the cultivated plots, where no cultivation has been practiced for at least the last 30 years. The reader is referred to the Methods sections of Figuerola et al., 2012, for a detailed description of these contrasting agricultural practices. Each treatment and depth were sampled in three replicates from 5 m^2^ quadrants separated by at least 50 m from each other. Soil samples were transported with dry ice to the laboratory, sieved through 4 mm mesh to remove roots and plant detritus, and processed 24 hr after collection. Total community RNA was extracted from 2 g of each replicate soil sample using the RNA PowerSoil^®^ Total RNA Isolation Kit (MO BIO) following the manufacturer's instructions. Total community DNA was extracted from 0.25 g of each replicate soil sample using FastDNASpin kit for soil (MP Biomedicals), in accordance with the manufacturer's instructions. The resulting RNA was treated with DNase I Amplification Grade (Invitrogen^™^). Quality and concentration of extracted nucleic acid were subsequently assessed by gel electrophoresis and spectrometry (Nanodrop, ThermoScientific).

### 2.2 Reverse transcriptase (RT)‐PCR amplification

cDNA was synthesized from 200 ng of RNA of each replicate soil using the SuperScript III reverse transcriptase (Invitrogen^™^) with three picomol of the universal primer *nif*H3 (Zani, Mellon, Collier, & Zehr, 2000). The rest of the reagents were added as per manufacturer's instructions. The final volume of the reaction mix was 20 μl. Reaction mixtures were incubated at 55°C for 50 min.

After reverse transcription, 2 μl (10 ng/μl) of the *nif*H‐cDNA was used for the first step of nested PCR as proposed by Yeager et al. (2004). For the second PCR slight modifications were incorporated. We used 2 μl of undiluted PCR product as template and primers included Roche 454 tag sequences (in bold) fused to the 5′ end (*nif*H11 5′**CACGACGTTGTAAAACGAC** GAY CCN AAR GCN GAC TC 3′ and *nif*H22 5′**CAGGAAACAGCTATGACC** ADW GCC ATC ATY TCR CC 3′). The PCR reactions were conducted in triplicate to minimize random PCR bias. Amplicons were purified by using NucleoSpin^®^ Gel Extract II kit (Macherey‐Nagel) and quantified using a Nanodrop Spectrophotometer. Replicates were pooled in equimolar concentrations in a single treatment library. Negative controls were applied in order to confirm that RT‐PCR results come from RNA template and not from possible genomic DNA contamination. Extracted RNA from all depth–treatment soil samples was treated with DNase I. After this treatment, 1 μl of RNA (200 ng/μl) was subjected to RT‐PCR reaction without reverse transcriptase and subsequently to *nif*H nested PCR. The DNA contamination was also monitored through agarose gel electrophoresis during the different experimental steps (Fig. S3).

### 2.3 *nif*H amplicon pyrosequencing and sequence processing

The diversity and structure of the active diazotrophic communities were evaluated by pyrosequencing analysis of the *nif*H gene. Each of the six RNA libraries (3 treatments × 2 depth soil) was labeled with a unique oligonucleotide barcode and pyrosequenced using 454 GS FLX technology. Pyrosequencing data were processed as previously described by Collavino et al. (2014). Briefly, short‐ and low‐quality sequences (Q value = 30), putative frame shifts, and chimeras were removed from the database. Remaining high‐quality sequences were clustered with OTUs defined at 98% amino acid sequence similarity, as discussed in Collavino et al., 2014. OTUs with at least three sequences were selected and their relative abundance was normalized using the subsampling‐based method described in mothur (http://www.mothur.org/wiki/Normalize.shared) prior to comparative analyses. OTU representative amino acid sequences along with sequences selected from the *nif*H reference database (http://wwwzehr.pmc.ucsc.edu/nifH_Database_Public/) were used to build protein phylogenetic trees.

The sequences obtained in this study have been deposited in the NCBI‐SRA (Sequence Read Archive) with the submission ID SRP029166 and BioProject ID PRJNA214426.

### 2.4 Taxa‐specific qPCR of *nif*H

Primers were designed to amplify the *nif*H sequences of two abundant phylotypes of the ADC classified within the subclusters 1A (related to *Geobacter*) and 1B (related to *Nostoc*). Target sequences were aligned and visually compared using the Jalview v2. The target OTUs 1A for phylotypes related to *Geobacter* (8, 22, 27, 53, 380, and 488) were amplified with GeoFw 5′ GCC AAR GCG CAG AAT ACG GT 3′ and the reverse primer *nif*H2 (Zehr & McReynolds, 1989). The OTU 1B (755) was amplified with cyanoFw 5′ TGG TAT CAT CAC CGC CAT CAA CTT C 3′ and cyanoRev 5′ GAA TTG GCA TAG CGA AAC CAC CG 3′. The amplicon size was 314 bp and 116 bp for phylotypes 1A and 1B, respectively. Theoretical calculations for primer concentrations and annealing temperatures (Ta) for each pair of primers were determined using the web tool OligoAnalyzer in IDT DNA (https://www.idtdna.com/calc/analyzer). Primers were experimentally evaluated through PCR amplification of DNA from *Geobacter* and *Nostoc*, as well as from other phylogenetic distant diazotrophs (e.g., *Herbaspirillum* sp., *Burkholderia* sp., *Azotobacter* sp., and *Gluconacetobacter* sp.). Amplification with cyanobacterial primers showed the expected amplicon size (116 bp) in soil samples as well as in *Nostoc* DNA (Fig. S4A). The PCR reaction consisted of 1 μl DNA (10 ng/μl), 0.2 μmol/L of each primer, 1.25 U Go Taq (Promega), Buffer Go Taq 10X, 2.5 mmol/L MgCl_2_ (Promega), and ultrapure water (Promega) up to 25 μl. Cycling conditions were 5 min at 95°C, followed by 30 cycles of 1 min at 94°C, 1 min at 60°C and 2 min at 72°C, and a final step of 10 min at 72°C. No amplification was detected in other phylogenetic distant diazotrophs under these PCR experimental conditions (Fig S4A).

The optimization of PCR conditions, annealing temperature, and the amount of template were necessary to improve efficiency and specificity for the amplification of *nif*H genes related to *Geobacter*. PCR reaction consisted of 1 μl DNA (5 ng, 10 ng, and 15 ng), 0.2 μmol/L of each primer, 1.25 U Go Taq (Promega), Buffer Go Taq 10X, 2.5 mmol/L MgCl_2_ (Promega), and ultrapure water (Promega) up to 25 μl. The cycling conditions were 5 min at 95°C, followed by 30 cycles of 1 min at 94°C, 1 min of Ta (57–58–59°C), and 2 min at 72°C, and a final step of 10 min at 72°C. The expected amplicon size of 314 bp was clearly visualized in the environmental samples with all the amounts of template tested, but with higher efficiency at 10 ng/μl of template (data not shown). Amplification was observed using 57°C and 58°C of Ta (Fig. S4B), whereas no amplification was detected in some environmental samples at 59°C (Fig. S4B, line 6). The amplification of G*eobacter* sp. DNA showed additional bands using 10 ng/μl or 15 ng/μl of template in all Ta evaluated (Fig. S4B, lines 13, 14, and 15). These spurious bands were eliminated by reducing the amount of template to 5 ng and using 57°C or 58°C of Ta (Fig. S4C). Therefore, *Herbaspirillum* sp., *Burkholderia* sp., *Azotobacter* sp., and *Gluconacetobacter* sp. were amplified using 5 ng of template and 57°C or 58°C of Ta. As shown in Fig. S4D, nonamplification was detected in the DNA tested at both Ta, except for *Gluconacetobacter* sp. which amplified at 57°C but not at 58°C of Ta. Finally, the PCR conditions used for the amplification of *nif*H gene with primers GeoFw‐*nif*H2 were 58°C of Ta using 5 ng/μl and 10 ng/μl for strain DNAs and environmental samples, respectively. It should be mentioned that GeoFw has only one A‐G primer–target mismatch at nucleotide position 8 with respect to *nif*H from *G*. *diazotrophicus* (Genbank accession AF105225), nevertheless non‐*nif*H gene amplification was detected from *G*. *diazotrophicus* DNA in the optimized PCR conditions (Fig. S4D). In addition, the specificity of the set GeoFw‐*nif*H2 primers in the environmental samples was analyzed by sequencing the amplified products. *nif*H amplicons generated from the different treatment and depth samples were pooled, an aliquot of 100 ng was purified using a commercial kit (NucleoSpin^®^ Extract II, Macherey‐Nagel) and cloned using the TOPO‐TA pCR2.1 kit (Invitrogen) according to the manufacturer's conditions. A total of 52 clones were sequenced using capillary‐based Sanger sequencing method. Nucleotides sequences were identified using blastX against NCBI database. The best match for all sequences corresponded to *nif*H sequences related to *Geobacter* species with identity values >97% (data not shown), which would indicate that using the GeoFw‐*nif*H2 primers in our environmental samples the largest proportion of amplicons corresponds to *Geobacter nif*H sequences. The sequences obtained were deposited in NCBI reservoir under GenBank accession numbers KY941188 to KY941239.

The relative abundance of the selected phylotypes was quantified by qPCR using as a template the DNA extracted from the same soil samples selected for the analysis of *nif*H‐RNA. The qPCR reactions contained 10 ng of soil DNA, 0.8 μl of each primer (5 μmol/L), 10 μl of 2× SYBR Green iCycler iQ mixture (Bio‐Rad), and water ultrapure for 20 μl final reaction volume. The reaction was carried out in the thermocycler Applied Biosystems 7,500 Real‐time PCR system (Applied Biosystems). The thermocycling program has the following parameters: initial denaturing at 95°C for 10 min, followed by 40 cycles of 95°C for 15 s, followed by an annealing step at 60°C (group 1B)/58°C (group 1A) for 20 s, and a final elongation step at 72°C for 20 s. Nontemplate controls were included in all runs. Fluorescence was measured at the end of each cycle. 16S rRNA gene abundances quantified by qPCR using universal primers 338F‐518R (Park & Crowley, 2005) were employed to normalize the *nif*H values between the different samples. The relative levels of the *nif*H were calculated using the tool GENORM (https://genorm.cmgg.be/). All qPCR reactions were run in duplicate with DNA extracted from two biological replicates, and the melting curve of each run was analyzed to ensure the proper development of amplification cycle.

### 2.5 Soil physical and chemical characterization

General soil properties were determined in crushed and 2 mm sieved samples (“fine earth”): pH (1:2.5 soil:water) by potentiometry, organic carbon (OC) by dry combustion method using a LECO CR12 19 equipment, and electric conductivity (EC) with a conductimeter. The cation exchange capacity (CEC) was measured at pH 7 with ammonium acetate by extraction with potassium chloride (Chapman, 1965). Particle size distribution was measured by means of the Robinson's pipette method for the clay and silt fractions and by sieving for the sand fractions (Soil Conservation Service, 1972). Total Nitrogen (TN) was measured by the Kjeldahl method (Bremner, 1996) and extractable phosphorous was determined by the method of Bray and Kurtz (1945).

Soil bulk density (BD) was determined by the core method (Blake & Hartge, 1986) using 138.5 cm^3^ volume cores (*n* = 9). Three samples were obtained for each depth at each subsampling area for the three treatments assessed (treatment *n* = 27). Aggregate stability was determined with fast immersion in water (slaking process) according to Le Bissonnais (1996). Thus, three undisturbed soil cores (6,000 cm^3^) were taken from the topsoil (0–15 cm) from each sampling unit. Results are presented as mean weight diameter (MWD).

The soil–water retention curve was measured in undisturbed soil samples (cores, 135 cm^3^) at matric suctions of 10, 30, 60, 90, 330, 2,000, and 15,000 cm in a pressure plate apparatus (Klute, 1986) with depths of 0–10 and 10–20 cm (*n* = 27). Water moisture and matric suction pairs were fitted to Van Genuchten (1980) water retention model, according to equation (1), considering the Mualem restriction m = 1‐1/n (Mualem, 1986). This procedure was done with RETC software (Van Genuchten, Leij, & Yates, 1991), using nonlinear least squares optimization, reaching in all cases parameters estimations above R^2^>0.96.

(1) $$\theta_{g}=\left(\theta_{\mathrm{gs}}-\theta_{\mathrm{gr}}\right){\left[1+{\left(\alpha h\right)}^{n}\right]}^{-m}+\theta_{\mathrm{gr}};h\geq 0$$

where θ_g_ (kg kg^−1^) is gravimetric water content, θ_*gs*_ (kg kg^−1^) is the saturated gravimetric water content, θ_gr_ (kg kg^−1^) is the residual gravimetric water content, θ (−) is the normalized water content, and α, n, and m are empirical curve‐fitting parameters.

Different pore sizes, >300 μm (P_Mac>300_) and <50 μm (P_Mic<50_) corresponding to large macroporosity and microporosity (Reynolds et al., 2002), respectively, were obtained with the fitted water retention curves (equation (2), (3)) considering Or and Wraith (2000) equation.

(2) $$P_{\mathrm{Mac}>300}=\theta_{s}-\theta_{\mathrm{Mac}\geq 300\;\mu m}\left(h_{\mathrm{Mac}\geq 300\;\mu m}=-0.1\;m\right);0\leq P_{\mathrm{Mac}}>300\leq \theta_{s}$$

(3) $$P_{\mathrm{Mic}<50}=\theta s-\theta_{\mathrm{Mac}\geq 50\;\mu m}\left(h_{\mathrm{Mac}\geq 50\;\mu m}=-0.6\;m\right);0\leq P_{\mathrm{Mic}<50}\leq \theta_{s}$$

where θ_*s*_ is 0 water potential (saturation).

For each sample, a pore volume distribution function was determined according to Reynolds, Drury, Tan, Fox, and Yang (2009) and Shahab, Emami, Hagnia, and Karimi (2013) with a further normalization of this function to allow the comparison between different soil samples. Pore volume distributions can be characterized using location and shape parameters (Reynolds et al., 2009), where the location parameters include the mode (D_mode_), median (D_median_), and mean (D_mean_) pore diameter values, and the shape parameters include standard deviation of porosity volume (Por. SD), skewness (asymmetry), and kurtosis (peakedness).

D_mode_ corresponds to the inflection point of the pore volume distribution function, being this location with the most frequent pore diameter value. Por. SD quantifies the size range of equivalent pore diameters, where Por. SD = 1 indicates pore diameter homogeneity while high Por. SD indicates an increase in equivalent pore diameters range. Skewness = 0 indicates a lognormal distribution. While negative values indicate high numbers of small pores, positive values indicates high number of large pores. Kurtosis = 1 corresponds to a lognormal distribution; values > 1 indicate a “leptokurtic” distribution which is more peaked in the center and more tailed in the extremes than the lognormal curve, whereas values < 1 indicate a “platykurtic” distribution which is less peaked in the center and less tailed in the extremes than the lognormal curve (Reynolds et al., 2009).

### 2.6 Statistical data analysis

The α‐diversity of the active diazotrophic community was evaluated by rarefaction curves with the rarefaction single Mothur command using resampling without replacement approach (Schloss et al., 2009). The estimators of richness (Chao1, S_Chao1_) and diversity Shannon–Wiener (H’) were calculated from the OTUs normalized abundance matrix using Past3 program (Hammer, Harper, & Ryan, 2009). To determine the significance of richness, diversity, and *nif*H relative abundance across treatment‐depth samples, one‐way and two‐way ANOVA models coupled with Tukey's multiple comparison of means were applied with the statistical package InfoStat version 2011 (Di Rienzo et al., 2008). For comparative analysis of the composition between samples (β‐diversity) two measures of dissimilarity were applied. Bray–Curtis measure (Bray & Curtis, 1957) was calculated from the abundance of *nif*H subclusters while weighted normalize UniFrac measure (Lozupone, Lladser, Knights, Stombaugh, & Knight, 2011) was applied on the abundance and phylogeny of the *nif*H OTUs. These measures were calculated using Past3 program and online Unifrac program (http://unifrac.colorado.edu/), respectively. Principal Coordinate Analysis (PCoA) was used to visualize the variation in ADC composition across treatment–depth samples.

Associations between physical and chemical soil variables (pH, Pe, TN, OC, CEC, BD, P_Mac>300_, P_Mic<50_, D_mode_, D_mean_, and Por. SD), estimators of richness and evenness (S_Chao1_ and H’, respectively), were assessed by principal component analysis (PCA) performed on the correlation matrix.

All location and shape of pore volume distribution curve variables and P_Mac>300_ were transformed (Log_10_) as they did not attain normality (Shapiro–Wilkis, *p* < .05). Kurtosis could not be normalized with standard methods therefore only descriptive data are presented.

## 3 RESULTS AND DISCUSSION

### 3.1 Diversity of the active diazotrophic community

We were able to synthesize and subsequently PCR amplify *nif*H‐cDNA from total RNA extracted from two soil depths (topsoil: 0–10 cm, subsurface soil: 10–20 cm). We assume that this cDNA represents the functional community of free‐living nitrogen‐fixing microorganisms, and hereafter we will refer to this as the active diazotrophic community (ADC). For each soil depth, the ADC was examined in two different no‐till agricultural managements (GAP and PAP for good and poor agricultural practices, respectively). Natural grassland close to the cultivated plots was used as reference (NE for natural environment). After sequencing and processing data, a total of 80 787 reads, comprising 28 171 protein‐encoding *nif*H genes were obtained and used to define 437 OTUs with a cutoff of 98% similarity at amino acid level. These *nif*H encoding sequences formed the *nif*H‐cDNA database and were used to calculate richness and diversity.

The ADC diversity was assessed using rarefaction curves and estimators of richness (S_Chao1_) and evenness (H’) (Fig. S1 and Table 1). According to S_Chao1_ the relative coverage across the six samples ranged between 66% and 100% (Table 1). Most of rarefaction curves nearly reached asymptote, indicating that a substantial part of the active diazotrophs diversity was recovered from our sampled soils. The evenness and richness of the ADC were both strongly affected by the agricultural use. It is the nondisturbed soil (NE) that showed the highest levels of diversity (H’) and richness (S_Chao1_) at both depths (Table 1). Two‐way analysis of variance (ANOVA) indicated a significant interaction effect between treatment and depth (*r* ^2^ = 1, *p *=* *1e^−4^ for H’ and *r* ^2^ = 0.98, *p *=* *1e^−4^ for S_Chao1_). Diversity in NE and GAP soils was higher in the first 0–10 cm than at 10–20 cm (*p *=* *3e^−4^ for NE depths and *p *<* *1e^−4^ for GAP depths), whereas in PAP the highest H’ was found in the 10–20 cm depth (*p *<* *1e^−4^).

**Table 1:** Sample coverage and diversity indices for *nif*H‐cDNA libraries

Depth (cm)/management	SeqN	SeqNn	Sobs	H′	S_Chao1_	R_Chao1_
0–10						
NE	3,983	3,151	231	3.96 f	245 d	94
GAP	6,265	3,159	67	1.12 c	67 b	100
PAP	4,997	3,159	20	0.11 a	27 a	74
0–20						
NE	3,335	3,159	166	3.32 e	183 c	91
GAP	4,463	3,159	33	0.37 b	50 ab	66
PAP	5,128	3,159	143	2.57 d	175 c	82
Management				***	***	
Depth				NS	NS	
Management × Depth				***	***	

The richness estimator (S_Chao1_) and Shannon's diversity index (H′) were calculated with OTUs defined at 98% amino acid sequence similarity**.** SeqNn, normalized number of sequences per sample; Sobs, detected number of operational taxonomic units (OTUs) at 2% distance level; RC, relative coverage calculated as OTU number divided by estimated richness (S_Chao1_). Different lowercase letters indicate statistical differences based on one‐way ANOVA (Tukey test, *p* < .05). Significant differences in the interaction (*^**^ *p *<* *.001). NS, not significant.

Therefore, these results suggest that ADC diversity and richness in soil are negatively affected by both the soil management practices studied, relative to the nearby nondisturbed soils. Moreover, the diazotrophic diversity seems to be influenced by depth albeit dependent of the agricultural soil use.

In addition, we compared the diversity of the ADC with the potential community previously described after pyrosequencing DNA‐*nif*H from the same site (Collavino et al., 2014). For this purpose, reads from cDNA of *nif*H were pooled across depths. The primers used for DNA and cDNA pyrosequencing were different, and we cannot rule out that primer bias could be affecting the results; however, both sets of primers targeted the same *nif*H conserved region. As shown in Fig. S2, no significant difference in H’ was found between the potential (DNA) and active (RNA) communities of uncultivated soils (*p *=* *.367). In cultivated soils, however, the diversity of potential community was significantly higher than the ADC (*p *<* *1e^−4^ and *p *=* *2e^−3^ for GAP and PAP, respectively). Moreover, a lower number of OTUs (229 in GAP and 173 in PAP) was observed in the cultivated soils of ADC compared to those observed in the potential community (361 in GAP and 381 in PAP). We have previously reported that richness and diversity of the potential diazotrophic community were not affected by soil management practices (Collavino et al., 2014). The diversity difference among potential and active diazotrophic community suggests that while no‐tillage management seems not to affect the presence of potential N_2_ fixers, but does influence *nif*H expression of some of them.

Fierer et al. (2012) compared microbial diversity of two soils, one with successional grassland and the other with agricultural use, and observed relative lower diversity in the agricultural site as compared to the grassland site. It is possible to speculate that soils having more plant diversity could promote higher bacterial diversity as in the case of NE with natural indigenous grasses, as compared with soils of either soybean–maize rotation (GAP) or soybean monoculture (PAP).

Eilers and colleagues evaluated the effect of soil depth on the structure of Bacteria and Archaea communities by 16S rRNA pyrosequencing, noting that diversity is greater in the first 10 cm compared to 10–25 cm and 175 cm, decreasing by 20% and 40%, respectively (Eilers, Debenport, Anderson, & Fierer, 2012). Similar results using techniques of DNA fingerprinting showed a decrease in bacterial diversity in deeper layers of soil (Agnelli et al., 2004; Fierer, Schimel, & Holden, 2003; Will et al., 2010). In addition, an increase in total bacteria diversity and transcription activity in the waste surface horizon compared with the organic horizon was found in coniferous forest soils over a period of decomposition of organic matter (Baldrian et al., 2012). Together, these studies indicate that bacterial community diversity tends to decrease in the lowest soil depths, which is also found in the case of the ADC examined in this work except for soil samples under PAP. As detailed later, richness and diversity were correlated with physical parameters related to pore type and stability (Figure 5). The topsoil of PAP showed the lowest structural condition mainly due to a decrease in their aggregate stability, which could be explaining the opposite trend of diversity in relation to depth observed in this treatment. Also, the excessive use of agrochemicals could be affecting the presence of certain groups (e.g., OTUs related to *Bradyrhizobium*, as detailed below) and thus decreasing diazotrophic topsoil diversity.

### 3.2 Comparison of diazotrophic communities across contrasting agricultural soil management and depth

**Figure 1:**
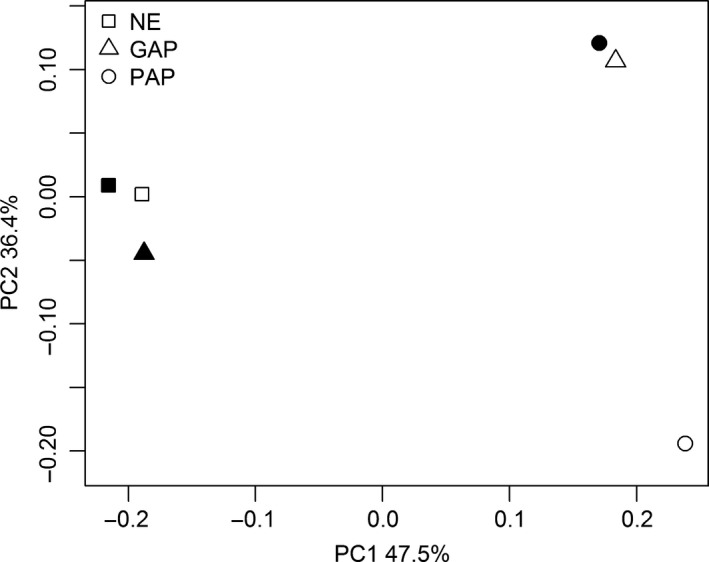
Relationship among diazotrophic communities examined by principal coordinate analysis (PCoA) using *weighted* UniFrac distance matrix. Closeness on the scatter plot indicates similarity between samples in terms of OTUs proportions and phylogeny. The communities under different agricultural use are coded by different symbols: square for NE, triangle for GAP, and circle for PAP. Depths 0–10 cm and 10–20 cm are, respectively, shaded in white and black

Patterns of ADC β‐diversity across all samples were examined using principal coordinate analysis (PCoA) with weighted UniFrac distances and Bray–Curtis dissimilarities. The results of both analyses showed similar ordinations and groupings between soil samples as well as a high percentage of total variance explained (PC1 47.5% for UniFrac and 55.8% for Bray–Curtis). As shown in Figure 1 for weighted normalized UniFrac analysis (Bray–Curtis‐based ordinations are not shown), PC1 and PC2 appear to group together the two NE depths and the 20 cm of GAP, whereas PAP 20 cm and GAP 10 cm group separately. PC1 and PC2 clearly put PAP 10 cm separate from all samples. This ordination shows that GAP diazotrophic communities have similarities with those of PAP and NE, while NE and PAP samples are distantly arranged between them. The PAP management, consisting in soybean monoculture and nonsustainable practices, has a noticeable effect on the ADC structure at both depths compared to that of soil under intensive crop rotation practice (GAP) and noncultivated soil (NE). Similarly, it has been shown by Rosa et al. (2014) that PAP management compared to NE presented an important effect on the size and the functioning of the denitrification community in Argentinean cropping fields. Moreover, PCoA plots show that samples from 0 to 10 cm are more dissimilar than samples from 10 to 20, given by the proximity in the plot. This is also reflected in the lower number of OTUs shared among all the 0–10 cm samples (3 OTUs) compared to those shared in the 10–20 cm samples (11 OTUs). The topsoil, expected to have more physical and chemical disturbances due to the agricultural practices, showed the most different diazotrophic community between treatments.

### 3.3 Composition of the active diazotrophic community of soil

The 437 distinct OTUs obtained from Pergamino soils were assigned into the four major clusters and the 17 subclusters that comprise the *nif*H phylogeny as previously defined by Zehr et al. (2003). The most abundant group across the entire *nif*H‐cDNA database was subcluster 1A (44.8%) composed by sequences related to *Anaeromyxobacter* and *Desulfuromonadales* (Table 2). Other dominant taxa were as follows: Alphaproteobacteria from subcluster 1K (29%) represented by rhizobiales sequences, Cyanobacteria from subcluster 1B (16.7%) related to the *Nostocales*, and *Verrucomicrobiales* and *Desulfovibrionales* from subcluster 3B (8%). The remaining *nif*H subclusters represented less than 1% of the total sequence count (Table 2).

**Table 2:** Composition of phylotypes in *nif*H‐cDNA amplified from Pergamino soils

	%			Group	Order^d^
	Sequence count^a^	OTU count^b^	Distribution^c^		
Subcluster					
1	0.1	0.5	33	Epsilon	*Campylobacterales*
1A	44.8	48.5	83	Delta	*Myxococcales* and *Desulfuromonadales*
1B	16.7	2.3	50	Cyanobacteria	*Nostocales*
1C	0.6	1.1	50	Firmicutes	*Clostridiales*
1E	0.2	0.7	33	Firmicutes	*Bacillales*
1G	0.05	0.2	50	Gamma	*Pseudomonadales* and *Enterobacteriales*
1J	0.4	1.1	83	Alfa and Beta	*Rhizobiales* and *Rhodospirillales*
1K	29.0	24.7	100	Alfa	*Rhizobiales*
1O	0.05	0.2	17	Gamma	*Chromatiales*
1P	0.2	1.1	67	Beta	*Rhodocyclales*
3B	8.0	19.5	67	Delta and Verrucomicrobia	*Desulfovibrionales* and V*errucomicrobiales*

a Percentage of sequences of *nif*H‐cDNA database assigned to each *nif*H subcluster.

b Percentage of OTUs classified in each *nif*H subcluster.

c Presence across the six samples analyzed (e.g., subcluster present in all samples shows a distribution of 100%).

d Orders closest to the predominant sequences observed in the subcluster.

**Figure 2:**
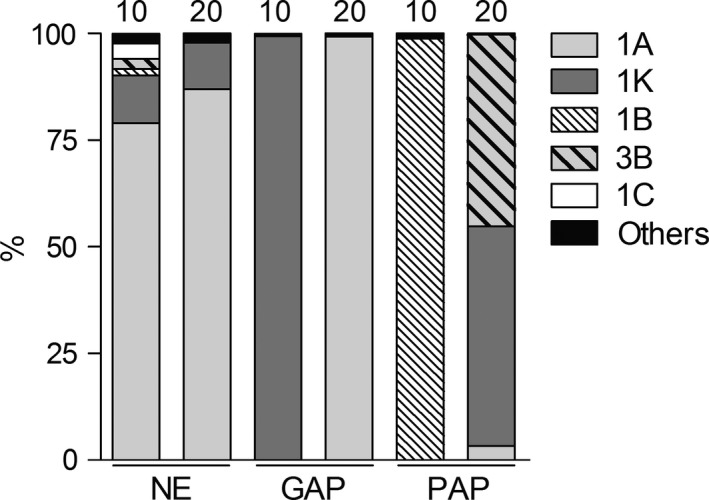
Distribution of the *nif*H subclusters across treatments and depths. Subclusters with a representation above 1% are shown, whereas subclusters with a representation <1% are shown together in “Others”. Different treatments are indicated as NE, natural environment, GAP, good no‐till agricultural practices, and PAP, poor no‐till agricultural practices. Depth 0–10 and 10–20 cm are labeled as 10 and 20, respectively

The diazotrophic community composition was mostly similar at both depths of the uncultivated soil (NE) as shown in Figure 1, in which populations that belong to subclusters 1A (79% to 87%) and 1K (11%) were highly represented (Figure 2). Also, the underrepresented subclusters (with < 1% of the total sequences) were mainly found at both depths of uncultivated soils. Contrarily, in cultivated soils the community composition showed strong differences in relation to soil depth and management practices. In GAP soils, subcluster 1K (99.4%) and 1A (99.3%) were dominated at the topsoil and subsurface, respectively (Figure 2). In PAP soils, subcluster 1B was predominant (98.8%) in the topsoil, whereas subclusters 1K (51.6%), 3B (45%), and 1A (3%) were found in the subsurface (Figure 2).

**Figure 3:**
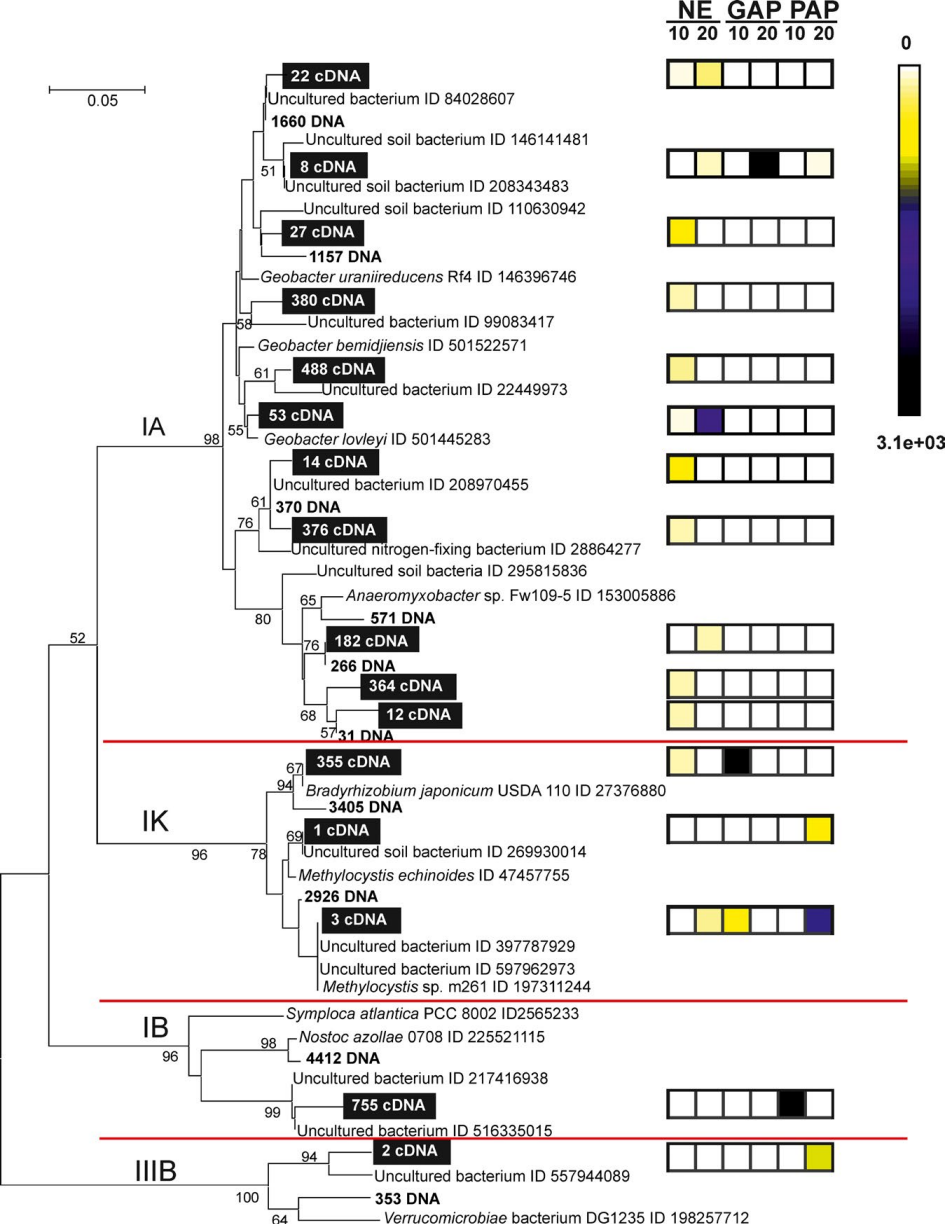
Neighbor‐joining phylogenetic tree of partial amino acid *nif*H sequences of the 16 most abundant OTUs. The abundant OTUs are identified with numbers within black boxes. OTUs in bold were obtained from DNA of Pergamino according to previous work described by Collavino et al. (2014). The diagram in the right margin showed the number of normalized *nif*H sequences assigned to each OTU in the different depth–treatment sample. Canonical *nif*H subclusters are indicated to the left in the figure. Only bootstrap values >50% are shown

Sixteen OTUs were represented by more than 100 sequences each and accounted for 53% of the total *nif*H‐cDNA database. These abundant OTUs were distributed among subclusters 1A (11 OTUs), 1K (3 OTUs), 1B (1 OTU), and 3B (1 OTU), as shown in Figure 3.

Six OTUs belonging to subcluster 1A, representing about 28% of the database, were related to *Geobacter* species (*G. uraniireducens* and *G. bemidjiensis*, and *G. lovely*). Noteworthy, except for OTU 8, all abundant 1A OTUs were found mainly in NE and showed a differential depth distribution (Figure 3). Particularly, OTU 8 highly represented in GAP 20 cm depth was closely related to *G*. *uraniireducens* and *nif*H clones reported as uncultured soil bacteria (Buckley et al., 2007; Hsu & Buckley, 2009). The sequences of these clones were obtained by an experimental approach using ^15^N‐stable isotopic probing (Buckley et al., 2007; Hsu & Buckley, 2009). As this approach relies on N‐fixation activity, and our study is based on cDNA analysis, altogether these findings suggest that populations closely related to anaerobic facultative *Geobacter* sp. could be important functional components in free‐living N_2_ fixation in soil.

The OTUs classified within subclusters 1K, 1B, and 3B were highly represented in cultivated soils and showed a differential distribution across depth (Figure 3). The 1K OTUs represented 25% of the *nif*H‐cDNA database and fell in two groups related to rhizobiales sequences. Particularly, OTU 355 more closely related to *B. japonicum* USDA 110, a broadly used strain in soybean commercial inoculants, was found mainly in the topsoil of GAP. Although, both GAP and PAP have soybean crop with *Bradyrhizobium* exogenous inoculation, the more stressing conditions by the use of agrochemical and nonnutrient replacement may affect the survival of *Bradyrhizobium* in PAP soil.

There is an increasing understanding that N_2_ fixation mediated by rhizobia is not limited to symbiotic association with leguminous plant host. Transcripts of *nif*H gene phylogenetically related to *Bradyrhizobium* and *Rhizobium* sp. have been found in endophyte community of rice and sugarcane roots (Burbano et al., 2011; Sessitsch et al., 2012). A high‐throughput study of the ecological profile of diazotrophic communities from soil lacking legumes revealed several *nif*H sequences related to *Bradyrhizobium* and *Rhizobium* (Wang et al., 2013). Wang and collaborates proposed that rhizobia could have a wide range of lifestyles, including that as a free‐living N_2_ fixer. Alternatively, it is possible to speculate that yet uncharacterized microorganisms may have received the *nif*H genes of rhizobia by horizontal transfer.

The remaining 1K OTUs related to the methanotrophic *Methylocystis* sp. were found mainly in the cultivated soils (Figure 3). This group has been reported as abundant soil diazotrophs (Collavino et al., 2014; Duc et al., 2009), likely due to its adaptive advantage to grow in soils with low organic matter content plus the dependence on the fermentation products of root exudates or crop retention residues (Buckley, Huangyutitham, Shi‐Fang, & Tyrrell, 2008).

The OTU 755 belonging to subcluster 1B, more similar to uncultured soil sequences and distantly related (83% similarity) to the heterocyst‐forming cyanobacteria *Nostoc azollae*, was the most abundant OTU in the database (16.4%) and found only in the topsoil of PAP (Figure 3). The cyanobacterial clade is a relevant diazotrophic group not only in aquatic environments (Bullerjahn & Post, 2014; Goebel, Edwards, Church, & Zehr, 2007; Zehr & McReynolds, 1989) but also in soil (Cavacini, 2001; Duc et al., 2009; Irisarri, Gonnet, & Monza, 2001; Teng et al., 2009; Yeager et al., 2007). Recently, the importance of this clade in N_2_‐fixation activity in Guerrero Negro intertidal microbial mats has been shown (Woebken et al., 2015). These surveys indicate the widespread distribution of N_2_‐fixing cyanobacteria and their active role in the functioning of various ecosystems including soils.

The OTU 2 classified in the subcluster 3B was mostly related to *nif*H sequences of noncultivated soil bacteria (Hoppe et al., 2014), but also to a certain extent to sequences from *Verrucomicrobiales* (82% similarity). This OTU was only found in the subsurface of PAP samples (Figure 3). The phylum Verrucomicrobia includes members that are ubiquitous and abundant in diverse habitats, including soil, water, and sediments (Kielak, Pijl, Van Veen, & Kowalchuk, 2008; Kielak et al., 2010; Sangwan, Chen, Hugenholtz, & Janssen, 2004), and their representatives are preferentially found in the 10–50 cm depth of some soils (Bergmann et al., 2011; Eilers et al., 2012).

The abundant OTUs in the *nif*H‐cDNA database showed various degrees of identity with GenBank *nif*H sequences, and the novelty of several phylotypes is highlighted, possibly as key players in the process of soil N fixation. We also identified phylotypes linked to particular treatments and/or depth (e.g., *Geobacter,* Cyanobacteria, and Verrucomicrobia).

### 3.4 qPCR of specific *nif*H phylotypes

In order to evaluate the data obtained from deep sequencing of *nif*H‐cDNA, the abundance of two particular members of the N_2_‐fixing microbial communities in Pergamino soils was assessed by qPCR. Primers were designed and preliminary experiments were performed to define conditions that specifically amplify *nif*H sequences related to *Geobacter* and Cyanobacteria, respectively (Fig. S4).

**Figure 4:**
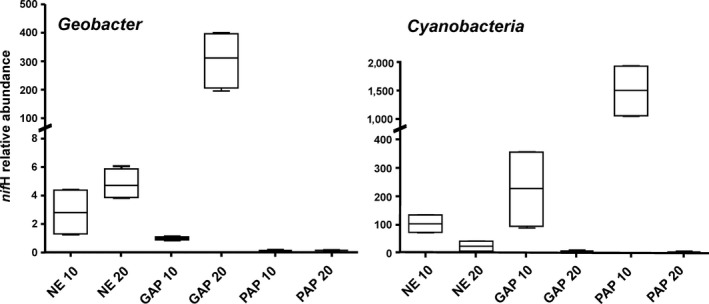
Quantitative abundance of *nif*H phylotypes related to *Geobacter* and Cyanobacteria in Pergamino soil samples as determined by qPCR. Level of *nif*H copies is shown as relative units normalized to 16S rRNA gene abundance. A value of 1 was assigned to the samples with the lowest detected value (GAP 0–10 cm and NE 10–20 cm to *Geobacter* and Cyanobacteria, respectively). Depth 0–10 and 10–20 cm are labeled as 10 and 20, respectively. Nondetectable *nif*H related to *Geobacter* and *Cyanobacteria* resulted in the PAP treatment and in the subsurface of cultivated soils, respectively. Boxes display the range of two biological and two technical replicates

As shown in Figure 4, the *nif*H gene copies of the phylotypes related to *Geobacter* was significantly different between NE and GAP (*p *=* *.009) and depths (*p *>* *.03). The pattern of relative abundance of *Geobacter nif*H resulting from qPCR analysis and the one revealed in the ADC were found similar to each other. Thus, it was higher in the subsurface of both NE and GAP soils with significant difference in the latter (*p *=* *1e^−4^), which suggests that this phylotype related to *Geobacter* may be adapted to grow at a deep layer of soil where anaerobic environment could facilitate expression of nitrogenase. It has been reported that members of the *Geobacter* clade are able to use the facultative anaerobic respiration (Lovley et al., 2011) as well as to express nitrogen‐fixation gene *nif*D in subsurface sediments (Holmes, Nevin, & Lovley, 2004). By contrast, the group was not detected in PAP samples (Figure 4). This treatment that has low nutrient addition could negatively affect the presence of this heterotrophic group.

Cyanobacterial *nif*H gene was detected in the topsoil of all treatments with higher value in PAP sample (*r* ^2^ = 0.85, *p *=* *2e^−4^), whereas it was only detected at lower abundance in the subsurface of NE (*p *=* *.009) (Figure 4). Occurrence of Cyanobacteria in the topsoil could be linked to the fact that this layer presents beneficial environmental conditions for photosynthetic activity and aerobic metabolism (Whitton & Potts, 2000).

Overall, these results together with those from *nif*H‐cDNA suggest that phylotypes related to *Geobacter* and Cyanobacteria could be considered important players of the free‐nitrogen fixation in agricultural soils.

### 3.5 Land‐use, edaphic properties, and active diazotrophic community

The physical and chemical characteristics of the land‐use types are presented in Table 3. No granulometric differences were observed across the soils examined. Tendency of OC was NE>PAP>GAP (Table 2). High OC at both sampling depths of NE (data not shown) could be explained by the lack of disturbance and the presence of active roots the year‐round (Novelli et al., 2011; Six, Elliott, Paustian, & Doran, 1998).

**Table 3:** Texture and chemical properties of soils for management treatments (0–20 cm)

Management	Granulometry						EC		pH		CEC		BS		OC		TN		EP	
	Clay		Silt		Sand		mS cm^−1^		1:2.5		cmol_c_ Kg^−1^		(%)		(%)		(%)		ppm	
	(<2 μm)		(2–50 μm)		(>50 μm)															
	g Kg^−1^																			
NE	218	0.6	623	0.2	15.9	0.4	0.36	0.03	5.7	0.1	18.9	0.9	72.8	2.8	1.8	0.2	0.19	0.05	3.6	1.7
GAP	228	1.5	602	1.5	16.9	0.3	0.39	0.02	5.5	0.1	17.1	1.0	71.6	2.1	1.3	0.1	0.14	0.02	10.5	8.2
PAP	211	1.6	643	0.9	14.6	0.7	0.26	0.04	6.2	0.0	18.0	0.7	80.3	3.8	1.6	0.0	0.14	0.01	1.5	0.8

CEC, Cation exchange capacity; EC, electrical conductivity; OC, total organic carbon; TN, total nitrogen; EP, extractable phosphorus, BS, Base saturation. Granulometric and chemical variables were not significantly different by depth; therefore, average values (0–20 cm) are presented. Treatments are labeled as: NE, natural environment; GAP, good no‐till agricultural practices; PAP, poor no‐till agricultural practices. Underlined values indicate standard error.

Location and shape porosity curve variables were affected by management practices, but not by depth (Table S1). Pore diameter values (D_mode_), statistical distribution of the pores (Por. SD), and the nonnormality pore size distribution (Skewness) were significantly higher in NE than cultivated soils (Table S1). Por.SD of this treatment largely exceeds the expected optimal ranges proposed by Shahab et al. (2013) indicating that diverse sizes of pore diameter are present in NE. Macroporosity (P_Mac>300_) showed a significant interaction between treatment and depth. In GAP and NE soils the higher values were observed in the first 0–10 cm, whereas in PAP the highest value was found in the 10–20 cm depth (Table S2). Bulk density (BD) was different between land‐use types and the highest values were found in the agricultural soils (Table S2). High BD values and the occurrence of platy structures (Kraemer et al., 2017) can cause worse porosity conditions in GAP and PAP compared to NE. Moreover, aggregate stability (fast water immersion) from NE was significantly higher (MWD: 2.00 mm) than the agricultural treatments (*p *<* *.05), whereas GAP (MWD: 0.50 mm) presented higher aggregate stability with respect to PAP (MWD: 0.38) without statistical significance.

**Figure 5:**
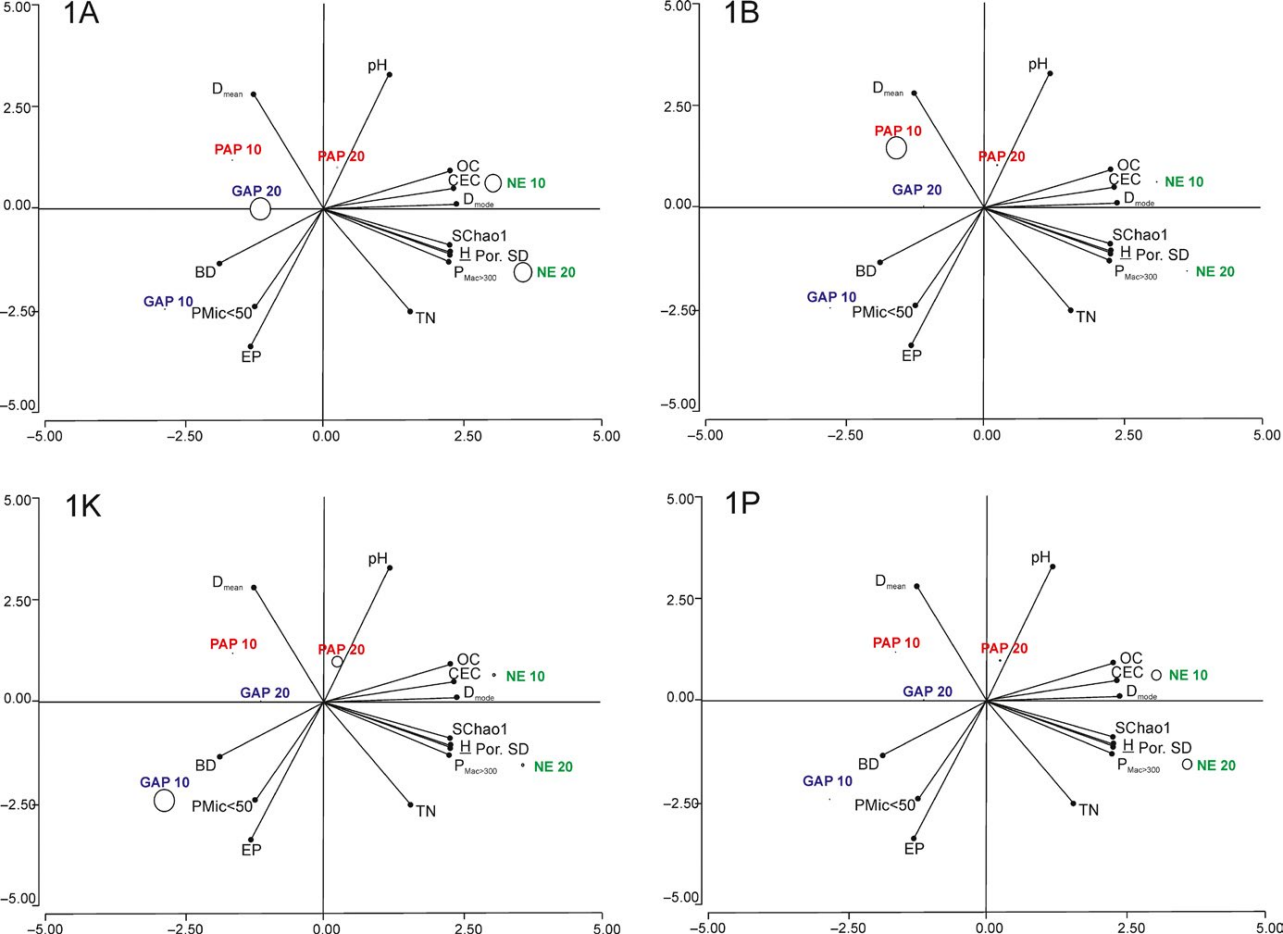
Proportion of *nif*H phylotypes related to diversity, treatment, depth, and soil physicochemistry. The proportions of subclusters 1A, 1B, 1K, and 1P are indicated by circle size at each sample point plotted on the first two principal components of the soil physicochemical parameters, richness, and diversity indexes (represented by lines). Component 1 (horizontal axis) covers 61.7% (mainly by Dmode: 0.34, CEC: 0.34, and H: 0.33) and component 2 (vertical axis) covers additional 20.1% of variation (mainly by pH: 0.48, Pe: ‐0.47) of the variance. Richness and diversity are represented by SChao1 and H, respectively. The different land‐type use is coded by different colors (green for NE, blue for GAP, and red for PAP)

The relationships between the diversity of the ADC and edaphic soil properties are shown in Figure 5. Diversity and richness appear to be mainly driven by several edaphic properties that characterize the NE soils. These properties include D_mode_, Por.SD, CEC, and OC. Diversity also shows significant correlations with hydrophobicity (Letey, 1969) (*r* = .9, *p *=* *.015), aggregate stability (*r* = .82, *p *=* *.04), and soil visual quality assessment (Ball, Batey, & Munkholm, 2007) (*r* = −.8, *p *=* *.05). Interestingly, a high proportion of subclusters 1A and 1P (one of the underrepresented subclusters) was found associated with NE soils conditions (Figure 5). Moreover, as mentioned previously, all the nonabundant *nif*H subclusters were mainly represented in NE soils (Figure 2). Diversity of bacterial communities as well as activity and microscale distribution are affected by pore structure (Carson et al., 2010; Chau, Bagtzoglou, & Willig, 2011; Nunan et al., 2003). In addition, more structured soil favors bacterial survival by offering them a higher diversity of physical habitats (Chenu & Cosentino, 2011).

Also, the correlation between diversity and OC has been found while examining the diversity of *nif*H‐DNA sequences from the same soils (Collavino et al., 2014). It has been found that OC increases the abundance, diversity, and activity of soil diazotrophic communities, which may be explained by the promotion of heterotrophic N_2_‐fixing bacteria in a niche with more available carbon (Billings, Schaeffer, & Evans, 2003; Huhe et al., 2014; Orr, James, Leifert, Cooper, & Cummings, 2011).

The topsoil of PAP was found related to higher average pore size (D_mean_) values in opposition to D_mode_ and P_Mac>300_, indicating an association with small diameter pores (Figure 5). Proportion of the cyanobacterial subcluster (1B) increased under this management (Figure 2 and Figure 5), suggesting that the niche occupied by phylotypes related to Cyanobacteria is characterized by strong fluctuation in water regime (oxic and anoxic environment). Water regime fluctuations are often affected by depth, and this condition and the great light exposure on the topsoil could be explaining the strong abundance differences in this subcluster observed throughout both sampling layers in PAP (Figure 2). Reports on the impact of soil physics in the cyanobacterial N_2_‐fixation activity are limited. A previous study reported a relationship among soil instability, water stress, and the predominance of *Nostoc* and *Scytonema* in biological soil crusts (Yeager et al., 2004). High D_mean_ value together with low OC and Por. SD (Figure 5) are indicators of poor soil structural quality and well‐sorted physical microenvironments. Accordingly, cyanobacterial *nif*H relative abundance was higher under cultivated treatments where OC and physical quality were lower (Figure 4 and Table 3). Moreover, PAP treatment has lower plant coverage compared to GAP and NE, which present winter cover crops or grassland, respectively (Figuerola et al., 2012). The low coverage could generate a greater sunlight exposure in PAP compared to the other topsoil samples. This together with low aggregate stability could explain the high representation of phylotypes related to Cyanobacteria in PAP topsoil (Figure 5). In addition, the abundance of this group was found negatively correlated with TN, OC, and EP (data not shown); which may indicate that Cyanobacteria have an ecological advantage in these limiting nutrient conditions.

The topsoil of GAP was associated with physical properties related to poor structural conditions, such as high proportion of micropores (P_Mic<50_) or “water storage pores” and high density (BD) (Figure 5). In loamy soils, as those of Pergamino, these physical parameters may create conditions for microaerophilic environments that allow N_2_ fixation by microaerophilic organisms such as *Bradyrhizobium* and the methanotrophic group *Methylosinus*/*Methylocystis*. Accordingly, the proportion of subcluster 1K, composed mostly by rhizobiales sequences, was found to be increased in the GAP topsoil sample (Figure 2 and Figure 5).

### 3.6 Concluding remarks

In recent years many high‐throughput studies have been reported on soil, which generally revealed a broad microbial diversity, some of them by using taxonomic markers such as 16S rRNA genes while others by examining functional genes such as the genes coding for denitrification, nitrification, nitrogen fixation, etc. However, these studies often do not associate the microbial data with the edaphic properties, land use, or agronomic practices; neither determined which components of the microbial community are indeed functionally active under agricultural conditions. In this work, the structure and diversity of the active diazotrophic community and its relation to physicochemical soil properties were assessed within a framework of a productive agricultural management system. Despite the limited number of soil samples analyzed, the deep pyrosequencing approach was useful to reveal underrepresented N‐fixing microorganisms and a high diversity of the ADC in soil samples. Indeed, the predominance of some phylotypes observed in the *nif*H‐cDNA data was validated by qPCR analysis.

The *nif*H‐cDNA database encompass 437 OTUs, whereas about 600 OTUS were identified in the DNA analysis previously reported (Collavino et al., 2014). This suggests that the ADC from Pergamino soils represents an important fraction of the potential diazotrophic community, although it cannot be ruled out that primer biases could be affecting the comparative analysis. The diversity and composition of the active diazotrophic community are influenced by management practices under no till, and this effect is in part related to the physical quality and organic carbon content. In soils with favorable structural conditions and higher OC, the N_2_ fixation could rely on a diverse community with a key role of 1A group, while poorer soil physical conditions drive the predominance of few groups, such as *Rhizobiales*, Cyanobacteria, and *Verrumicrobiales*, depending on management/depth condition. BNF is a complex cellular process as a large number of genes need to be activated in order to have a functional nitrogenase enzyme, energetically requires about 16 ATP molecules per mole of reduced nitrogen, and anaerobic or microaerobic environment to protect nitrogenase of irreversible damage. Therefore, it is expected that soil properties such as physical structure and organic carbon level may influence biological nitrogen fixation by specific taxonomic groups of diazotrophs.

The high representation of *nif*H phylotypes related to *Geobacter* and Cyanobacteria given at both potential and active levels provides solid bases to consider them as key players of free‐nitrogen fixation in agricultural soils. The occurrence of these groups was affected by management and depth conditions. For instance, the abundance of *nif*H gene related to *Geobacter* was found reduced in soils under intensive monoculture, which showed the lowest structural condition, whereas cyanobacterial *nif*H level was higher in the topsoil of this treatment.

This study shows the view of a single point in time of a particular soil type, therefore further temporal studies will help to determine how diazotrophic communities change over time and what soil properties and types of land use may drive the structuring of specific populations. Our results suggest strong associations of *nif*H phylotypes with soil properties and management practices, highlighting the importance of linking microbial community characterization with soil metadata to gain insight into the ecology of free‐living diazotrophs in soils.

## CONFLICT OF INTEREST

None declared.
