# Supplementary figures and images for: Analysis of nifH‐RNA reveals phylotypes related to Geobacter and Cyanobacteria as important functional components of the N2‐fixing community depending on depth and agricultural use of soil
Source: Microbiologyopen. 2017 Aug 1;6(5):e00502. doi: 10.1002/mbo3.502 (PMC5635172; doi:10.1002/mbo3.502)

Figure S1

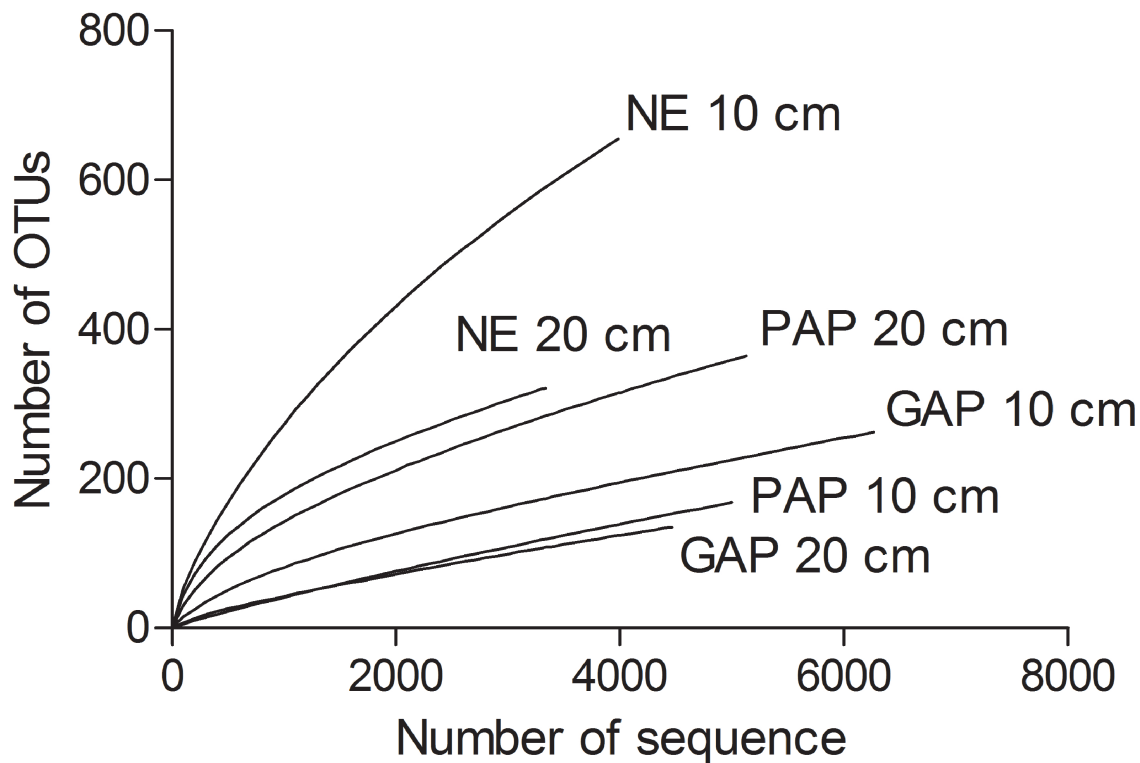

Supplement: Supplementary file 1 [file MBO3-6-na-s001.pdf]

# Figure S2

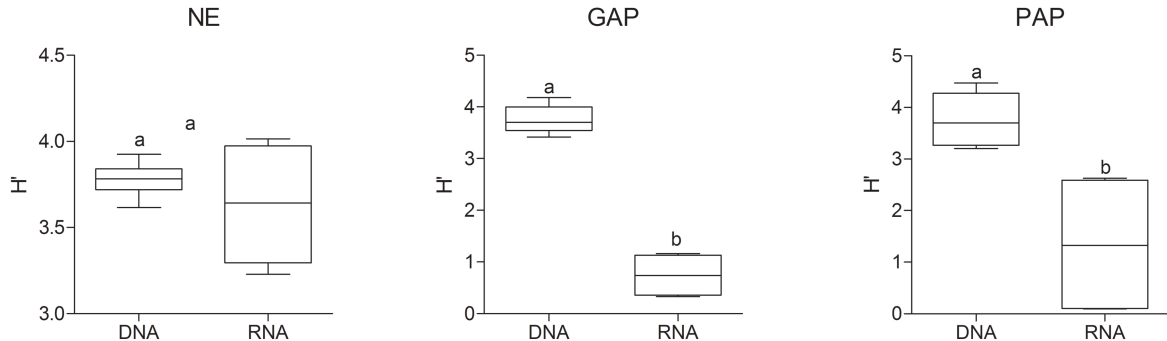

Supplement: Supplementary file 2 [file MBO3-6-na-s002.pdf]

Figure S3

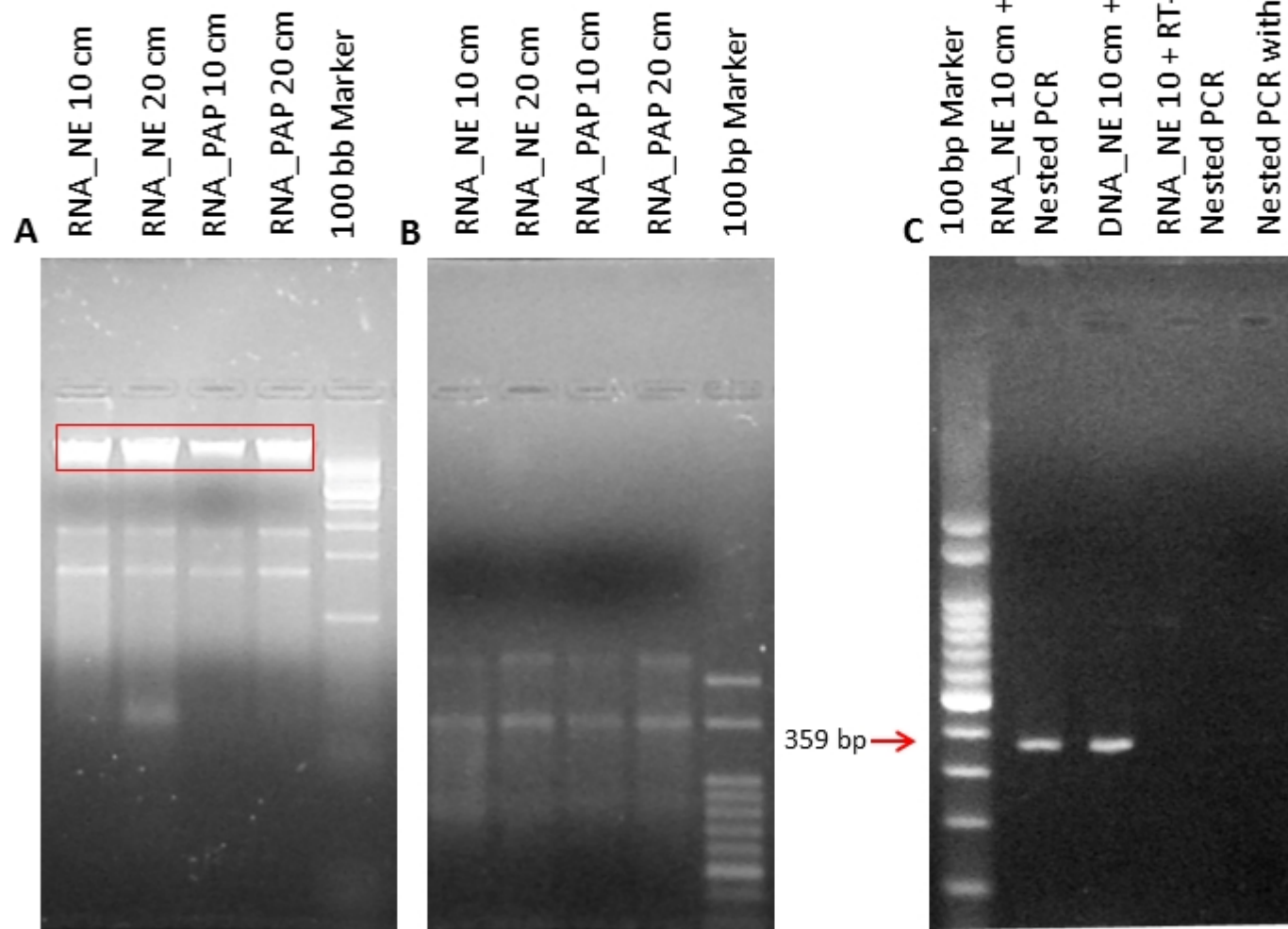

Supplement: Supplementary file 3 [file MBO3-6-na-s003.pdf]

Figure S4

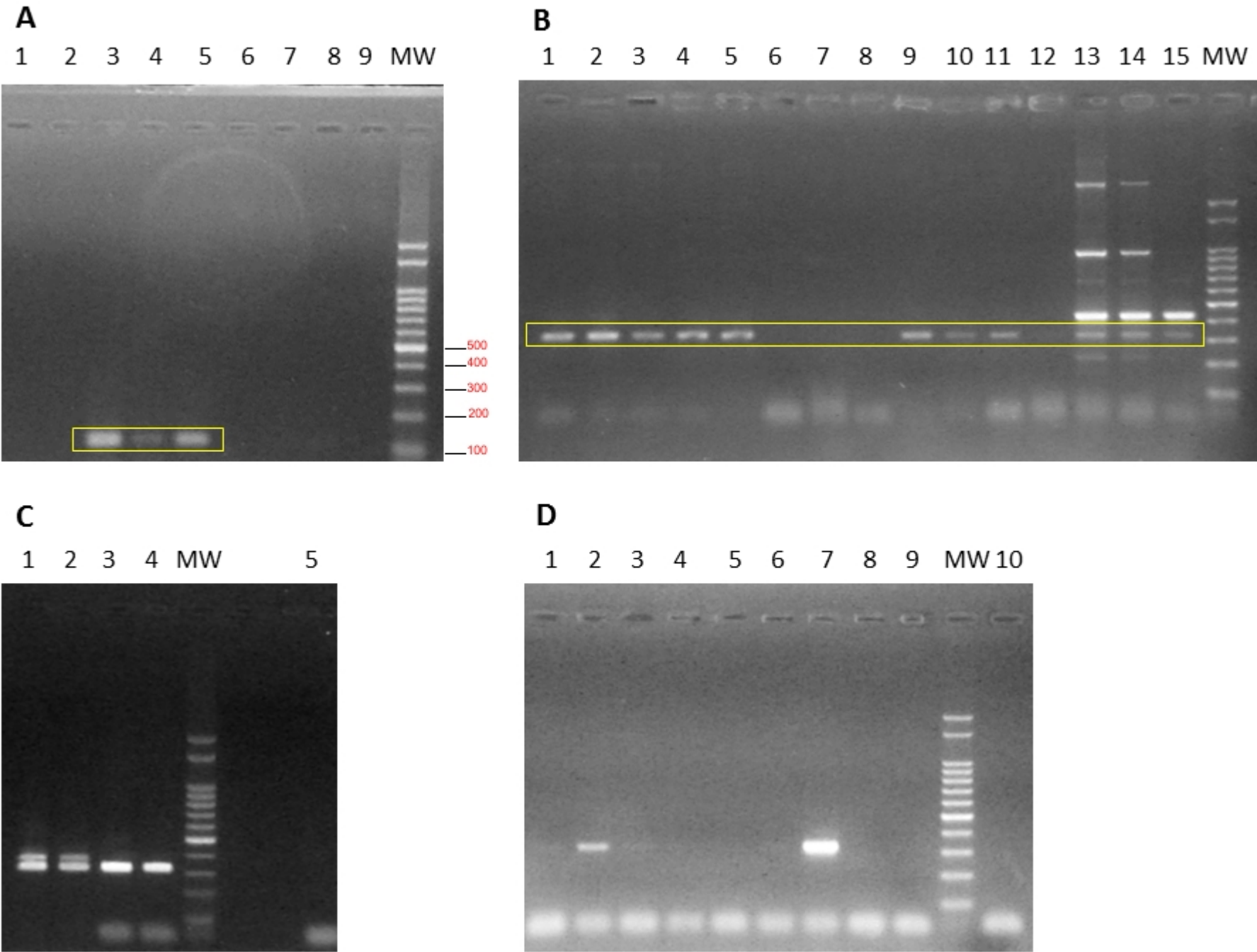

Supplement: Supplementary file 4 [file MBO3-6-na-s004.pdf]
