# Supplementary material for: Analysis of nifH‐RNA reveals phylotypes related to Geobacter and Cyanobacteria as important functional components of the N2‐fixing community depending on depth and agricultural use of soil
Source: Microbiologyopen. 2017 Aug 1;6(5):e00502. doi: 10.1002/mbo3.502 (PMC5635172; doi:10.1002/mbo3.502)
Supplement: Supplementary file 5 [file MBO3-6-na-s005.docx]

**Table S1**

| **Depth (cm)/**  **management** | | ***Location variables*** | | | | | | | |  | ***Shape porosity curve variables*** | | | | | | | |
| --- | --- | --- | --- | --- | --- | --- | --- | --- | --- | --- | --- | --- | --- | --- | --- | --- | --- | --- |
|  |  | **D_mode_**  **(μm)** | |  | **D_median_**  **(μm)** | |  | **D_mean_**  **(μm)** | |  | **Por. SD**  **(-)** | |  | **Skewness**  **(-)** | |  | **Kurtosis**  **(-)** | |
| 0-10 | NE | 133.6 | 28.9 | a | 10.6 | 16.3 | A | 6.6 | 11.1 | B | 72462.8 | 120283.45 | A | -0.42 | 0.05 | A | 1.13 | 0.02 |
|  | GAP | 23.4 | 17.5 | d | 4.6 | 7.5 | BA | 3.35 | 5.7 | AB | 1889.6 | 2431.4 | B | -0.40 | 0.055 | B | 1.14 | 0.01 |
|  | PAP | 31.2 | 7.1 | cd | 11.3 | 7.8 | B | 7.95 | 7.5 | A | 46.55 | 53.75 | B | -0.32 | 0.085 | B | 1.15 | 0.01 |
| 10-20 | NE | 74.7 | 32.9 | ab | 1.5 | 1.2 |  | 0.3 | 0.3 |  | 64489.3 | 109570.8 |  | -0.44 | 0.02 |  | 1.13 | 0.01 |
|  | GAP | 27 | 9.6 | cd | 9.6 | 4.3 |  | 5.9 | 2.9 |  | 20.6 | 3.4 |  | -0.33 | 0.01 |  | 1.16 | 0.01 |
|  | PAP | 53.9 | 0.1 | bc | 15.6 | 1.3 |  | 8.6 | 1.0 |  | 28.8 | 4.1 |  | -0.34 | 0.01 |  | 1.15 | 0.01 |
| Management | | *** | | | * | | | * | | | *** | | | * | | | - | |
| Depth | | ns | | | ns | | | ns | | | ns | | | ns | | | - | |
| Management x  depth | | * | | | ns | | | ns | | | ns | | | ns | | | - | |

Different lowercase letters indicated statistical differences corresponding to the interaction management x depth. Different uppercase letters indicates statistical differences corresponding to management factor (0-20 cm).
