# Supplementary material for: Analysis of nifH‐RNA reveals phylotypes related to Geobacter and Cyanobacteria as important functional components of the N2‐fixing community depending on depth and agricultural use of soil
Source: Microbiologyopen. 2017 Aug 1;6(5):e00502. doi: 10.1002/mbo3.502 (PMC5635172; doi:10.1002/mbo3.502)
Supplement: Supplementary file 6 [file MBO3-6-na-s006.docx]

**Table S2.**

| **Depth (cm)/ management** | | **P_Mac_ >300 μm** | |  | **P_Mic_ <50 μm** | |  | **BD** | |  |
| --- | --- | --- | --- | --- | --- | --- | --- | --- | --- | --- |
|  |  | **m^3^ m^-3^** | | | | | **g cm^-3^** | | | |
| **0-10** | NE | 0.085 | 0.011 ^Ɨ^ | a | 0.405 | 0.040 |  | 1.25 | 0.10 | |
|  | GAP | 0.021 | 0.007 | bcd | 0.479 | 0.015 |  | 1.45 | 0.06 | |
|  | PAP | 0.016 | 0.003 | cd | 0.432 | 0.024 |  | 1.38 | 0.03 | |
| **10-20** | NE | 0.045 | 0.008 | ab | 0.414 | 0.005 |  | 1.14 | 0.08 | |
|  | GAP | 0.008 | 0.003 | d | 0.395 | 0.023 |  | 1.27 | 0.04 | |
|  | PAP | 0.031 | 0.003 | abc | 0.428 | 0.009 |  | 1.35 | 0.04 | |
| Management | | *** | | | ns | | * | | | |
| Depth | | ns | | | ns | | ns | | | |
| Management x Depth | | ** | | | ns | | ns | | | |
